# Supplementary material for: Overall success rate of permanent teeth pulpotomy using ProRoot MTA: A systematic review and meta-analysis of randomized clinical trials
Source: PLoS One. 2025 Apr 10;20(4):e0320838. doi: 10.1371/journal.pone.0320838 (PMC11984715; doi:10.1371/journal.pone.0320838)
Supplement: S1 Table — (DOCX) [file pone.0320838.s001.docx]

**Supplementary table 1.** Search strategy in each database.

| **Database** | **Search strategy** | **Findings** |
| --- | --- | --- |
| PubMed | #1: ((ProRoot MTA[MeSH Terms]) OR (ProRoot MTA) OR (Pro Root MTA) OR (pro-root MTA) OR (Proroot MTA) OR (proroot MTA) OR (mineral trioxide aggregate[MeSH Terms]) OR (mineral trioxide aggregate)) | 3195 |
|  | #2: ((pulpotomy[MeSH Terms]) OR (pulpotomy) OR (vital pulp therapy)) | 3243 |
|  | #3: ((permanent teeth) OR (dentition, permanent[MeSH Terms]) OR (secondary dentition)) | 21788 |
|  | **#1 AND #2 AND #3** | **166** |
| Cochrane Library | #1: "ProRoot MTA" OR "Pro Root MTA" OR "pro-root MTA" OR "Proroot MTA" OR "proroot MTA" OR "mineral trioxide aggregate" in Title Abstract Keyword | 421 |
|  | #2: pulpotomy OR "vital pulp therapy" in Title Abstract Keyword | 636 |
|  | #3:  "permanent teeth" OR "permanent dentition" OR "secondary dentition" in Title Abstract Keyword | 689 |
|  | **#1 AND #2 AND #3** | **41** |
| Scopus | #1: TITLE-ABS-KEY("ProRoot MTA") OR TITLE-ABS-KEY("Pro Root MTA") OR TITLE-ABS-KEY("pro-root MTA") OR TITLE-ABS-KEY("Proroot MTA") OR TITLE-ABS-KEY("proroot MTA") OR TITLE-ABS-KEY("mineral trioxide aggregate") | 3540 |
|  | #2: TITLE-ABS-KEY(pulpotomy) OR TITLE-ABS-KEY(vital pulp therapy) | 3065 |
|  | #3: TITLE-ABS-KEY("permanent teeth") OR TITLE-ABS-KEY("permanent dentition") OR TITLE-ABS-KEY("secondary dentition") | 13498 |
|  | **#1 AND #2 AND #3** | **135** |
| Web of Science | #1: TS=("ProRoot MTA" OR "Pro Root MTA" OR "pro-root MTA" OR "Proroot MTA" OR "proroot MTA" OR "mineral trioxide aggregate") | 3569 |
|  | #2: TS=(pulpotomy OR "vital pulp therapy") | 1217 |
|  | #3: TS=("permanent teeth" OR "permanent dentition" OR "secondary dentition") | 8063 |
|  | **#1 AND #2 AND #3** | **160** |
| Embase | #1: 'proroot mta'/exp OR 'proroot mta'/exp OR 'Pro Root MTA'/exp OR 'pro-root MTA'/exp OR 'Proroot MTA'/exp OR 'proroot MTA'/exp OR 'mineral trioxide aggregate'/exp OR 'mineral trioxide aggregate' | 8508 |
|  | #2: 'pulpotomy'/exp OR pulpotomy OR 'vital pulp therapy' | 1569 |
|  | #3: 'permanent teeth'/exp OR 'permanent teeth' OR 'permanent dentition'/exp OR 'permanent dentition' OR 'secondary dentition'/exp OR 'secondary dentition' | 10792 |
|  | **#1 AND #2 AND #3** | **97** |
| Science Direct | #1: "ProRoot MTA" OR "Pro Root MTA" OR "pro-root MTA" OR "Proroot MTA" OR "proroot MTA" OR "mineral trioxide aggregate" | 12196 |
|  | #2: pulpotomy OR "vital pulp therapy" | 2273 |
|  | #3: “permanent teeth" OR "permanent dentition" OR "secondary dentition" | 18695 |
|  | **#1 AND #2 AND #3** | **190** |
