# Supplementary figures and images for: Overall success rate of permanent teeth pulpotomy using ProRoot MTA: A systematic review and meta-analysis of randomized clinical trials
Source: PLoS One. 2025 Apr 10;20(4):e0320838. doi: 10.1371/journal.pone.0320838 (PMC11984715; doi:10.1371/journal.pone.0320838)

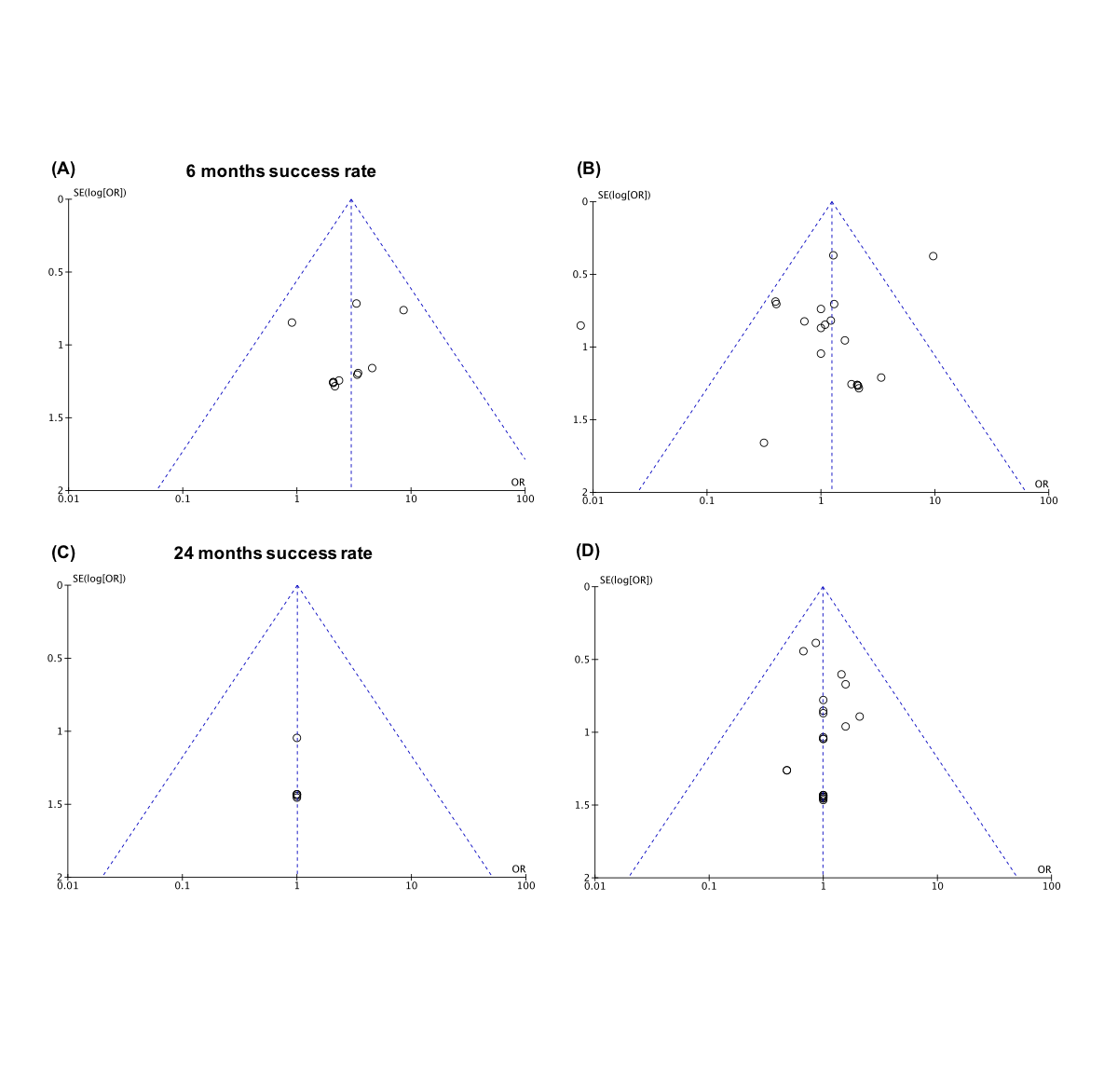

Supplement: S1 Fig — Funnel plots for the meta-analyses: (a) success rate at 6-month follow-up; (b) Success rate at 12-month follow-up; (c) success rate at 24-month follow-up; (d) Annual Failure Rate. (PNG) [file pone.0320838.s003.png]

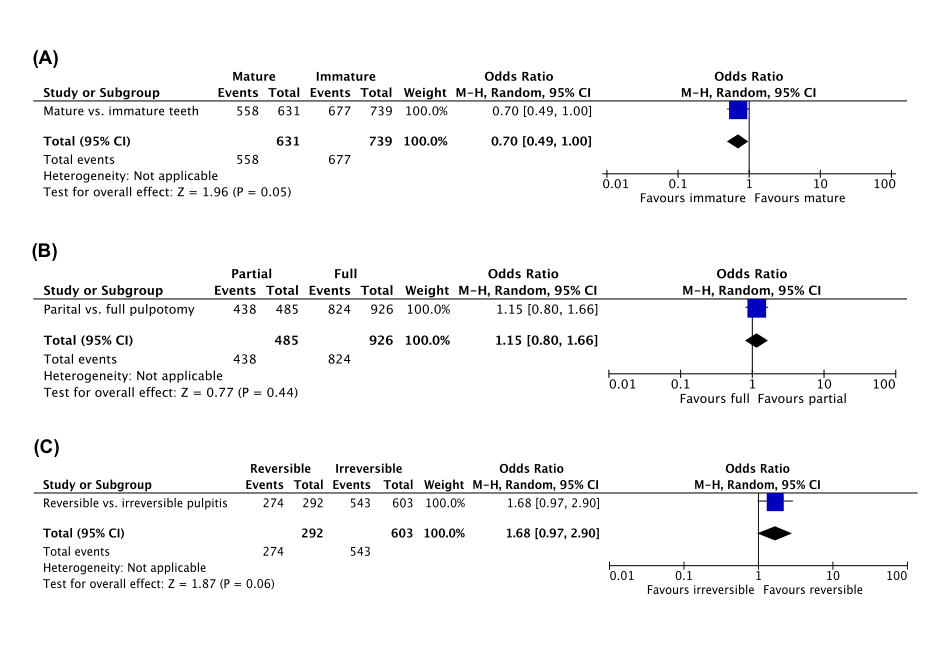

Supplement: S2 Fig — The forest plots indicated a statistically significant difference between (a) mature and immature teeth. In contrast, there was no significant difference observed in the comparisons of (b) partial and full pulpotomy and (c) teeth with reversible and irreversible pulpitis. (PNG) [file pone.0320838.s004.png]
