# Supplementary material for: Overall success rate of permanent teeth pulpotomy using ProRoot MTA: A systematic review and meta-analysis of randomized clinical trials
Source: PLoS One. 2025 Apr 10;20(4):e0320838. doi: 10.1371/journal.pone.0320838 (PMC11984715; doi:10.1371/journal.pone.0320838)
Supplement: Attachment — Submitted filename: Rebuttal letter.docx [file pone.0320838.s006.docx]

Dear Editor,

This manuscript was previously submitted to this journal (**PONE-D-24-28836; Overall success rate of permanent teeth pulpotomy using ProRoot MTA: a systematic review and meta-analysis of randomized clinical trials**) but was not accepted due to a concern related to the literature search completed over a year ago. The search was updated, and we are now submitting a new version of the manuscript. Follows the as

*Editor*: In the case of your submission, we are concerned that the literature review was completed in February 2023. Systematic and scoping reviews should provide an up-to-date overview of the research topic covered. Since the literature search was completed over a year ago, we are concerned that this study does not meet this requirement. As a result of these cannot consider the manuscript for publication at PLOS ONE at this time. However, if you are able to address the concerns discussed above, we may be able to consider a revised version of the manuscript.

R: Thank you for the consideration. We acknowledge your concern about the timeliness of the literature review, which was indeed completed in February 2023. To address this issue, we have conducted an updated literature search through August 2024. This update has allowed us to incorporate four new studies into the review, ensuring that our manuscript now provides a current and comprehensive overview of the research topic. We appreciate the opportunity to revise our submission and hope that the updated version meets the standards of PLOS ONE.
