# Supplementary material for: Overall success rate of permanent teeth pulpotomy using ProRoot MTA: A systematic review and meta-analysis of randomized clinical trials
Source: PLoS One. 2025 Apr 10;20(4):e0320838. doi: 10.1371/journal.pone.0320838 (PMC11984715; doi:10.1371/journal.pone.0320838)
Supplement: Attachment — Submitted filename: Response to the reviewers.docx [file pone.0320838.s007.docx]

**Dear Dr. Mohmed Isaqali Karobari**

**Academic Editor of PLOS ONE**

On behalf of co-authors, I am submitting the revised version of the manuscript PONE-D-24-38286 entitled *Overall success rate of permanent teeth pulpotomy using ProRoot MTA: a systematic review and meta-analysis of randomized clinical trials* to the PLoS One Journal. We would like to express our gratitude to you and the reviewers for their meticulous and insightful review of our work. Considering their valuable suggestions and comments, we have made some revisions to the manuscript. To clearly highlight these changes, we have marked them in blue throughout the document. We appreciate the opportunity to contribute to the PLoS One Journal and look forward to your favorable decision.

***Reviewer #1***

*I thank the Editorial Board of PLoS One for the opportunity to review the manuscript titled "Overall success rate of permanent teeth pulpotomy using ProRoot MTA: a systematic review and meta-analysis of randomized clinical trials." I commend the authors for their efforts on the manuscript so far. However, the article has significant limitations that cannot be addressed through revision, which prevents me from recommending its publication.*

Response: We thank Reviewer #1 for their thorough review and constructive comments on our manuscript. Below, we address each concern point by point, providing clarifications and justifications for our methodology and approach.

1. *Despite the efforts to meta-analyze the data, the data handling was not appropriate. First, a fixed-effect analysis assumes that all studies evaluated a common true effect and that the differences among the results are due only to chance or sampling error. However, upon reviewing the included studies, there are potential sources of discrepancies, whether due to diagnostic criteria, sample characteristics, the pulpotomy protocol applied, or even the potential biases identified by the RoB assessment.*

Response: We thank the reviewer concern. We also acknowledge the reviewer’s observation regarding the fixed-effect model. We chose this model because the statistical heterogeneity observed in our analyses was minimal, with I² values ranging from 0% to 20%. This indicator supports the appropriateness of a fixed-effect model in this context. Moreover, random-effects models were used in subgroup analyses where heterogeneity was higher. Importantly, this approach was conducted following the advice of our Biostatistical Advisor, who recommended using fixed-effect models for overall analyses with low heterogeneity and random-effects models for subgroup analyses to ensure methodological accuracy. Our approach aligns with established meta-analytic practices and ensures that the data handling reflects the characteristics of the included studies.

2. *The subgroup analysis does not align with the stated objective; a meta-regression would be more appropriate.*

Response: We also thank the reviewer for this concern. Although we understand the reviewer comment, we opted for subgroup analyses rather than meta-regression due to the limited number of included studies and the lack of sufficient covariates to perform a reliable meta-regression. Subgroup analyses allowed us to explore variations in success rates across key factors while maintaining statistical robustness. Conducting meta-regression with sparse data could risk overfitting and lead to unreliable conclusions, which we aimed to avoid. This approach was also recommended by our Biostatistical Advisor, who emphasized that subgroup analyses were the most suitable method given the data characteristics and the study design.

3. *Furthermore, treatment-related protocols were not even considered by the authors, except for the biomaterial used. While I understand this is the primary focus of the study, the pulpotomy technique is far more comprehensive, and its outcome is not solely dependent on the capping biomaterial used.*

Response: We thank the reviewer for this comment which is interesting indeed. While we agree that pulpotomy outcomes are influenced by multiple factors, our study's primary focus was on evaluating the success rate of ProRoot MTA as a capping material. However, we did consider and discuss other treatment-related factors, such as the type of pulpotomy (partial or full), root maturity, and pulpal diagnosis, in our subgroup analyses. A more comprehensive evaluation of additional treatment protocols (such as hemostatic agents, operator expertise) could be an avenue for future research, as detailed in the end of our discussion section.

4. *In addition, there is a clear lack of novelty in the review, and the data duplicates findings from other recently published reviews with a more focused research question. Other reviews have investigated the outcomes of pulpotomy in various aspects: partial vs. total, immature vs. mature teeth, irreversible vs. reversible pulpitis, or even according to the etiology of the indication.*

Response: We thank the reviewer for this comment. In our point of view, the present systematic review provides a unique contribution by exclusively focusing on ProRoot MTA, the most extensively studied MTA brand, and consolidating evidence from randomized clinical trials. While other reviews have explored broader or more specific aspects of pulpotomy, none have comprehensively synthesized RCT data on ProRoot MTA’s overall success rate in permanent teeth across various contexts. This focused approach ensures clinically relevant insights for practitioners using this specific biomaterial. Furthermore, we explicitly acknowledge in the Introduction section the existence of prior systematic reviews and discuss their limitations, such as selective inclusion criteria and narrower scopes. Our work builds upon these prior efforts to address these gaps and provide a comprehensive and clinically valuable synthesis of the available evidence. However, in order to avoid disappointing the reviewer, we have added it now to a new limitations sentence in the Discussion.

5. *Moreover, the findings of this review have already been confirmed in the literature, where the success rate of pulpotomy using ProRoot MTA is significantly higher than when using calcium hydroxide.*

Response: We have appreciated the reviewer concern. In our perspective, while the higher success rate of ProRoot MTA compared to calcium hydroxide is documented, our study extends this knowledge by presenting an updated and rigorous meta-analysis of 26 RCTs. Additionally, we evaluated success rates at multiple follow-up periods (6, 12, and 24 months), assessed the annual failure rate, and conducted subgroup analyses, offering a more granular understanding of ProRoot MTA’s performance in different clinical scenarios.

6. *Please refer to (1) Silva EJNL, Pinto KP, Belladonna FG, Ferreira CMA, Versiani MA, De-Deus G. Success rate of permanent teeth pulpotomy using bioactive materials: A systematic review and meta-analysis of randomized clinical trials. Int Endod J. 2023 Sep;56(9):1024-1041. doi: 10.1111/iej.13939. (2)* *Li W, Yang B, Shi J. Efficacy of pulpotomy for permanent teeth with carious pulp exposure: A systematic review and meta-analysis of randomized controlled trials. PLoS One. 2024 Jul 5;19(7). doi: 10.1371/journal.pone.0305218.*

Response: We appreciate this recommendation. The study (1) was already part of the bibliographic reference list, while the study (2) has now been added.

***Reviewer #2***

*The submitted paper, titled "Overall Success Rate of Permanent Teeth Pulpotomy Using ProRoot MTA: A Systematic Review and Meta-Analysis of Randomized Clinical Trials," aimed to evaluate the success rate of pulpotomy in permanent teeth using ProRoot MTA through a systematic review and meta-analysis. This review is highly relevant to the field of Endodontics and adheres to established guidelines.

Overall, the paper is well-written. This reviewer has only minor suggestions to enhance its readability.*

Response: We thank Reviewer #2 for their thorough review and constructive comments on our manuscript. All requested changes were performed as suggested by the reviewer.

*Minor comments:

- Page 2. Abstract: Replace "RevMan 5.3 and R software" with "RevMan 5.3 and R softwares."*

Response: Considering that “software” is an uncountable noun, the plural should be software. Therefore, in this specific point we do not made any modification.

*- Page 5. Line 4: Correct "MTA been established" to "MTA has been established."*

Response: Thank you! We have changed.

*- Page 5. Line 8: Include the city, state, and country of Dentsply Tulsa Dental.*

Response: It was added as suggested by the reviewer.

*- Page 6. Line 4 (Search strategy): The phrase "The search was performed without any filters, language restrictions, or August 2024" is incomplete and requires clarification.*

Response: Thank you! We have changed!

*- Page 12. Line 2: Replace "evolved" with "involved."*

Response: Thank you! We have changed!

*- Page 12. Line 8: Use the abbreviation AFR (Annual Failure Rate), as it was already defined earlier.*

Response: Thank you! We have changed!

*- Page 16: Revise "the remaining nine studies were categorized as having a low risk of bias" to reflect the correct number of studies, which is likely 12.*

Response: Thank you! It was changed.

*- Page 16. Line 3 (Meta-analysis): Use the abbreviation AFR instead of the full term Annual Failure Rate.*

Response: Thank you! We have changed!

*- Page 17. Line 1: Change "The assessment of 23 studies" to "The assessment of 26 studies," as the correct number is 26.*

Response: In fact for this specific assessment 23 studies were evaluated. Please check figure 4.

*- Page 17. Line 1: Use AFR for consistency instead of spelling out Annual Failure Rate.*

Response: Thank you! We have changed!

*- Page 17. Line 9: The phrase "Random-effect models were employed as heterogeneity could not be established" appears incomplete and needs clarification or additional context.*

Response: Thank you! We have changed!

*- Page 19. Line 5: Replace "randomized clinical trials was to assess" with "RCTs was to assess."*

Response: Thank you! We have eliminated RCT and used randomized clinical trial in the entire manuscript.

*- Page 19. Line 7: Use AFR instead of spelling out Annual Failure Rate.*

Response: Thank you! We have changed

*- Page 19. Line 8: Correct the Annual Failure Rate to 8%, as the reported value is incorrect.*

Response: Thank you! We have changed

*- Page 19: Revise "In this review, it was observed that permanent teeth of younger patients with an open apex displayed notably higher success rates in comparison to mature teeth, respectively." The word "respectively" seems misplaced and should be removed or rephrased for clarity.*

Response: Thank you! It was removed.

***Reviewer #3***

*Thank you for the opportunity to review the manuscript titled “Overall success rate of permanent teeth pulpotomy using ProRoot MTA: a systematic review and meta-analysis of randomized clinical trials.”

I am pleased to inform you that I found the manuscript to be exceptionally well-written, with a clear and concise presentation of the research. The authors have done an excellent job in articulating their findings and supporting them with robust evidence.*

*I have no recommendations for revisions or improvements, as the manuscript meets the high standards expected for publication in PLOS One.*

Response: We thank Reviewer #3 for their thorough review and constructive comments on our manuscript

***Reviewer #4***

*The article present relevant information for Endodontic practice and it was well designed. This article will show that MTA is still relevant and that bioceramic cements are not as essential as they are trying to make them out to be.*

Response: We thank Reviewer #4 for their thorough review and constructive comments on our manuscript
